# Strengthening health systems in communities: the experiences of Amref Health Africa

**DOI:** 10.11604/pamj.supp.2016.25.2.11262

**Published:** 2016-11-26

**Authors:** Githinji Gitahi

**Affiliations:** 1 Group Chief Executive Officer, Amref Health Africa, Nairobi, Kenya

**Keywords:** Africa, disease burden, health systems, MDG, knowledge generation

## Editorial

**Figure:**
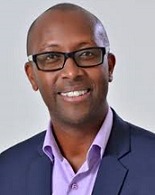


Africa is grappling with a set of challenges including; a multiple disease burden, malnutrition, illiteracy, environmental degradation, inequality, inequity, debilitating poverty situation and governance of the available resources among others. For sustainable development to be realised so that Africa breaks out of the vicious cycle of disease, it is important for the world and Africa in particular to seek for evidenced based solutions. In response to this need Amref Health Africa in its ten-year corporate strategy for 2007-2017 focused on “Putting African Communities First” with the goal of advancing health through catalysing an evidence-based movement aimed at reducing the gap between communities and the rest of the health system. The organization would partly implement this component of the strategy by use of health systems research approach for policy and practice.

Prioritising the strengthening of health systems in communities is one deliberate effort that would help achieve universal health coverage. The local and country level programming should be aligned to the global health objectives such as summarized during the era of Millennium Development Goals (MDGs) [1] and currently as the sustainable development goals (SDGs [2]). Most of the evidence presented in this supplement were experienced during the MDG era and thus focus on reducing child mortality (MDG4); improving maternal health (MDG5); combating HIV/AIDS, malaria and other diseases (MDG6); and ensuring environmental sustainability (MDG7).

As efforts are made to align evidence to the global health objectives, it is important to always take advantage of working with governmental and inter-governmental health agencies to contribute to generation of evidence in the African context to the global health agenda. Amref Health Africa as an organization contributes to health development in Africa, specifically on the improved health for mothers and children – notwithstanding that fathers should not be left behind. Organisational visions such as “lasting health change in Africa” can only be achieved in an environment of evidence based generation.

As an organisation Amref Health Africa recognises the importance and power of knowledge generation and translation as a process for policy and practice influence at local, national and global levels. Evidence based policy making in efforts to strengthening health systems and improving health outcomes is paramount. It is partly for this reason that the first Amref Health Africa conference was held in November 2014, with the next in March 2017 as Africa Health Agenda International Conference 2017. During such conferences researchers, policy makers and academicians gather to share experiential knowledge generated with a view of searching for solutions to health challenges in Africa. The majority of papers published in this Pan-African Medical Journal supplement were presented during the first Amref Health Africa Conference for accelerated dissemination of knowledge and search for solutions in health systems strengthening. We appreciate the efforts of all authors. Let’s join hands as we together search for solutions.
